# Child Deaths by Gun Violence in the US During the COVID-19 Pandemic

**DOI:** 10.1001/jamanetworkopen.2022.25339

**Published:** 2022-08-04

**Authors:** Pablo A. Peña, Anupam Jena

**Affiliations:** 1 Kenneth C. Griffin Department of Economics, University of Chicago, Chicago, Illinois; 2 Department of Health Care Policy, Harvard Medical School, Boston, Massachusetts; 3 Department of Medicine, Massachusetts General Hospital, Boston; 4 National Bureau of Economic Research, Cambridge, Massachusetts

## Abstract

This cross-sectional study examines gun-related deaths during the early stages of the US COVID-19 pandemic among children aged 0 to 17 years.

## Introduction

In addition to direct effects on health, the COVID-19 pandemic has had indirect effects through stress and economic hardships imposed by mitigation efforts such as lockdowns. For children, the indirect health outcomes may be comparable with direct effects, because SARS-CoV-2–related morbidity and mortality are concentrated at older ages.

One potential indirect health outcome for children is gun-related deaths owing to increased psychological strain in families in combination with school closures, which lead to greater time spent by children at home, where guns are often kept. Although studies have documented increased gun-related deaths during the pandemic, only few studies have considered children and those focused on early stages of the pandemic.^1,2,3,4^

## Methods

This cross-sectional study analyzed gun-related deaths of children between January 1, 2014, and December 31, 2022, from the Gun Violence Archive (GVA), a repository of gun violence collected from more than 7500 law enforcement, media, government, and commercial sources (eMethods in Supplement).^5^ The study used publicly available data and was thus exempt from human subjects review at Harvard Medical School, and it followed the STROBE reporting guideline.

We plotted 28-day moving averages of the count of children (aged 0 to 17 years) killed, comparing mid-March 2020 onwards to the trend in prior years to account for seasonal variation in shootings. In addition, we conducted an event study regression analysis with 2922 daily observations that modeled the daily count of child gun-related deaths as a function of a cubic time-trend and adjustments for month and day of week, assuming a pandemic period beginning March 16, 2020 (eMethods in the Supplement).

We conducted subgroup analyses according to child age, median household income in the Census tract where a death occurred (using the 2019 American Community Survey), and the percentage Black or Hispanic population in the Census tract (analysis conducted because the health and economic impact of the pandemic has varied by race). We used a significance threshold of *P* < .05 using a 2-sided test. Stata version 15.1 (StataCorp) was used for analyses from March to April 2022.

## Results

Overall, 8044 shootings were analyzed and involved 8477 children killed. Of these 8477 children, 1888 (22.3%) were aged 0 to 11 years and 6589 (77.7%) were 12 to 17 years; 1657 (19.6%) were girls, 6676 (78.8%) were boys, and 144 (1.7%) had unknown gender; and 4914 (58.0%) were from a high-minority area (defined as having more than 50% Black or Hispanic population). An increase in deaths was observed from March 16, 2020, onwards (Figure), with an estimated 1.12 (95% CI, 0.70-1.53) additional children killed per day, corresponding to an estimated 733 (95% CI, 462-1003) additional children killed over the study period.

**Figure.**
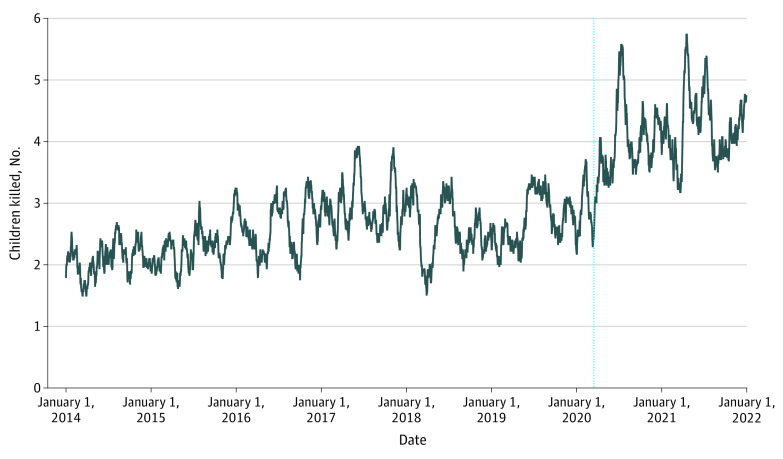
Daily Number of Children Killed in Shootings in the US Figure presents 28-day moving averages. The moving average includes fewer than 28 but at least 14 days at the start and the end of the period covered. Children included were aged 0 to 17 years. The vertical line indicates March 16, 2020. Source: Gun Violence Archive.

The US Centers for Disease Control and Prevention (CDC) reports that 752 children died from COVID-19 from January 1, 2020, to December 31, 2021,^6^ implying that our estimated increase in child gun-related deaths was comparable with COVID-19–related deaths in children. Increases in gun-related deaths were concentrated in areas with low median income or a high percentage of Black or Hispanic population, suggesting family and neighborhood socioeconomic conditions may be important mediators. They were also greater among children aged 12 to 17 years (Table). Findings were robust to analyses that modeled linear, quadratic, and quartic time trends.

**Table.** Estimated Change in the Daily Number of Children Killed in Shootings After March 15, 2020

Areas analyzed	No. of children killed in shootings	Change in mortality, additional daily No. of children killed in shootings (95% CI)	
		Cubic time trend	Quartic time trend
All areas	8477	1.12 (0.70 to 1.53)	1.18 (0.75 to 1.60)
Low-income areas^a^	6557	1.14 (0.77 to 1.51)	1.23 (0.85 to 1.60)
High-income areas^a^	1905	−0.02 (−0.21 to 0.16)	−0.05 (−0.25 to 0.16)
Low-minority areas^b^	3556	0.39 (0.13 to 0.66)	0.45 (0.17 to 0.72)
High-minority areas^b^	4914	0.72 (0.41 to 1.04)	0.73 (0.41 to 1.05)
Shooting victim age, y			
0-11	1888	0.38 (0.18 to 0.57)	0.40 (0.20 to 0.61)
12-17	6589	0.74 (0.39 to 1.10)	0.77 (0.41 to 1.14)

^a^ High-income (low-income) areas were defined as Census tracts where median annual household income was above (below) $60 000, which is an approximation of the median annual household income in the US in the period of study.

^b^ High-minority (low-minority) areas were defined as Census tracts where more (less) than 50% of the population was Black or Hispanic.

## Discussion

A comparison of gun-related deaths with CDC-reported COVID-19-related deaths suggests that the direct mortality effects of SARS-CoV-2 on children may be similar in magnitude to indirect outcomes mediated by gun violence. Factors that may have caused the observed increase in gun-related deaths among children are unknown and include psychological and economic strain due to the pandemic as well as greater time spent at home due to school closures. These findings highlight the potential importance of indirect health outcomes in children during the COVID-19 pandemic. Study limitations include analysis of a large gun violence database and confounding due to the study’s observational design. Given the coverage of the GVA in comparison with the CDC data, our figures may be underestimates.

## Notes

Supplement. **eMethods.** Supplemental Methods

## References

[1] Ashby MPJ. Initial evidence on the relationship between the coronavirus pandemic and crime in the United States. Crime Sci. 2020;9(1):6. doi:10.1186/s40163-020-00117-6 32455094 PMC7233195

[2] Peña PA, Jena A. Mass shootings in the US during the COVID-19 pandemic. JAMA Netw Open. 2021;4(9):e2125388. doi:10.1001/jamanetworkopen.2021.25388 34529068 PMC8446816

[3] Donnelly MR, Grigorian A, Swentek L, . Firearm violence against children in the United States: trends in the wake of the COVID-19 pandemic. J Trauma Acute Care Surg. 2022;92(1):65-68. doi:10.1097/TA.0000000000003347 34932041 PMC8677489

[4] Cohen JS, Donnelly K, Patel SJ, . Firearms injuries involving young children in the United States during the COVID-19 pandemic. Pediatrics. 2021;148(1):e2020042697. doi:10.1542/peds.2020-042697 33850026

[5] GVA. Gun Violence Archive. Accessed April 28, 2022. https://www.gunviolencearchive.org/

[6] Centers for Disease Control and Prevention. Provisional COVID-19 Deaths by Sex and Age. Published 2022. Accessed April 28, 2022. https://data.cdc.gov/NCHS/Provisional-COVID-19-Deaths-Focus-on-Ages-0-18-Yea/nr4s-juj3/data

