# Supplementary material for: Child Deaths by Gun Violence in the US During the COVID-19 Pandemic
Source: JAMA Netw Open. 2022 Aug 4;5(8):e2225339. doi: 10.1001/jamanetworkopen.2022.25339 (PMC9353595; doi:10.1001/jamanetworkopen.2022.25339)
Supplement: Supplement. — eMethods. Supplemental Methods [file jamanetwopen-e2225339-s001.pdf]

Supplemental Online Content

Peña PA, Jena A. Child deaths by gun violence in the US during the COVID-19 pandemic. *JAMA Netw Open.* 2022;5(8):e2225339.
doi:10.1001/jamanetworkopen.2022.25339

eMethods. Supplemental Methods

This supplemental material has been provided by the authors to give readers additional information about their work.

eMethods. Supplemental Methods

Data. We analyzed data on firearm injuries and deaths from the Gun Violence Archive, a registry that utilizes "automated queries, manual research through over 7,500 sources from local and state police, media, data aggregates, government and other sources daily." Although the Gun Violence Archive is one of few, publicly available, up-to-date sources on firearm injuries and deaths in the U.S., these data may be incomplete to the extent that they partially rely upon shootings reported in the media, where previous research has demonstrated that selective reporting of firearm injuries may occur based on victim characteristics (see, e.g., Kaufman EJ, Passman JE, Jacoby SF, et al. Making the news: Victim characteristics associated with media reporting on firearm injury. Prev Med. 2020;141:106275. doi:10.1016/j.ypmed.2020.106275). Issues of completeness of these data, however, would not be expected to vary before and after an assumed start date of the Covid-19 pandemic, thereby permitting use of these data to study the longitudinal question of changes in child shootings following the start of the pandemic.

Nonetheless, we conducted analyses to assess reliability of the Gun Violence Archive (GVA) data. Specifically, we compared the monthly number of children killed according to the Gun Violence Archive with information from the CDC on firearm deaths among children (0-17 years old), which is available monthly up to December 2020 (CDC WONDER, https://wonder.cdc.gov/controller/saved/D77/D277F530). The correlation between the two monthly time series was 0.90. According to CDC data, in the period from April to December of 2020 there was an increase of 481 in the number of children killed by firearms relative to the period from April to December of 2019. The corresponding increase in the GVA data was 340 (71% of the CDC figure), suggesting that the estimated increase in children killed according to the GVA data may understate the true increase.

Statistical Analysis. We estimated the following multivariable linear regression model in our baseline analysis:

$$y_t = \theta_m + \eta_d + \beta x_t + \gamma t + \delta t^2 + \eta t^3 + \varepsilon_t \qquad (1)$$

Where y_t is the number of children injured or killed on date t, θ_m is a month-of-year fixed effect that accounted for within-year fluctuations in the number of children injured or killed that could be associated with seasonal events like weather, school breaks, etc. The fixed effects were estimated with separate indicator variables for each month (January, February, March, etc.). η_d is a day-of-week fixed effect that accounted for within-week fluctuations in the number of children injured or killed that could be associated with different patterns across days (e.g., weekdays vs weekends). The fixed effects were

estimated with separate indicator variables for each day of the week (Sunday, Monday, Tuesday, etc.). x_t is an indicator variable that was equal to zero for dates before March 16, 2020, and equal to one for dates on or after March 16, 2020. β is the parameter of interest and is interpreted as the average increase in the daily number of children injured or killed associated with the pandemic. The term $\gamma t + \delta t^2 + \eta t^3$ represents a cubic trend in the number of children injured or killed over the entire period of analysis, from January 2014 to December 2021. Such a trend could be increasing, decreasing or flat, depending on the data. Lastly, ε_t is the error term. In sensitivity analyses, additional models were estimated using different types of time trends (linear, quadratic, and quartic).

To estimate the number of children killed above the expected trend, we used the fitted values according to model (1) for the period from March 16, 2020 to December 31, 2021, and compared them to the fitted values assuming x_t equal to zero.

Subgroup analysis. Three subgroup analyses were conducted. First, shooting incidents were categorized according to the age of the victims based on information from the Gun Violence Archive: 0-11 and 12-17 years of age. Separate analyses were conducted for each of those categories.

Additional subgroup analyses were performed by linking data from the Gun Violence Archive to Census tract level demographic and income data. Specifically, incidents recorded by the Gun Violence Archive include address. The address information was used to generate geographic coordinates, locate the Census tract where each shooting incident took place, and link it with information from the 2019 American Community Survey on median household income and racial or ethnic composition. Out the 28,633 shooting incidents that involved children injured or killed, over 99% were successfully linked to a Census tract with information on median household income or racial/ethnic composition.

Therefore, second, shooting incidents were categorized based on the median annual household income of the Census tract where they occurred: high-income areas corresponded to Census tracts with a median annual household income in 2019 above 60,000 dollars (which is an approximation of the median annual household income in the US in the period of study), and low-income areas corresponded to Census tracts with a median annual household income in 2019 of up to 60,000 dollars.

Third, shooting incidents were also categorized based on the racial or ethnic composition of the Census tract where they took place: high-minority areas corresponded to Census tracts where more than 50% of the population in 2019 was Black or Hispanic, and low-minority areas corresponded to Census tracts where up to 50% of the population in 2019 were Black or Hispanic.

© 2022 Peña PA et al. *JAMA Network Open.*
